# Adolescents’ Interest in Quitting Nicotine or Cannabis E-Cigarettes

**DOI:** 10.1177/00099228251355748

**Published:** 2025-07-20

**Authors:** Vira Pravosud, Pamela M. Ling, Bonnie Halpern-Felsher, Valerie Gribben

**Affiliations:** 1 Center for Tobacco Control Research and Education, University of California San Francisco, San Francisco, CA, USA; 2 Center for Data to Discovery and Delivery Innovation, San Francisco VA Health Care System, San Francisco, CA, USA; 3 Northern California Institute for Research and Education, San Francisco, CA, USA; 4 Division of General Internal Medicine, Department of Medicine, School of Medicine, University of California San Francisco, San Francisco, CA, USA; 5 REACH Lab, Division of Adolescent Medicine, Department of Pediatrics, Stanford University, Palo Alto, CA, USA; 6 Department of Pediatrics, University of California San Francisco, San Francisco, CA, USA

## Introduction

Prevention and cessation of nicotine or cannabis electronic cigarette (e-cigarette) use among youth remains one of the key public health priorities due to high rates of adolescent use^1,2^ and associated negative health effects.^3^ However, little is known about adolescents who are willing to quit vapor products and reasons they have not stopped using nicotine or cannabis e-cigarettes. The present study compared California middle and high school students, who reported use of nicotine and/or cannabis e-cigarettes during COVID-19 Shelter-in-Place orders (hereafter “Shelter-in-Place”), by interest in quitting nicotine or cannabis e-cigarettes and reasons why they have not quit. Given the popularity and high prevalence of social media use, online platforms provide promising opportunities for cessation of nicotine or cannabis e-cigarette use among young people.^4^ Thus, we also examined adolescents’ interests in Instagram-based cessation programs.

California was the first US state to legalize medicinal cannabis in 1996 for people 18 years and older with a medical provider’s recommendation and adult recreational use in November 2016 for people 21 years and older, with first legal sales starting in January 2018.^5,6^ Despite age restrictions for adult use, there has been an increase in cannabis use among California adolescents after legalization,^7^ and especially among young people who use e-cigarette products.^8,9^ Our study capturing data during the time of the pandemic complements existing research and informs prevention efforts regarding cessation of e-cigarette use in youth.

## Methods

This was a cross-sectional study of California adolescents surveyed on Qualtrics between August 2020 and March 2021.^10^ SIS International Research recruited adolescents by reaching out to parents on SIS research panels and by posting the screener questionnaire online. Respondents were eligible if they were (1) 13 to 18 years old (SIS verified by supporting documentation), (2) a middle or high school student in California before Shelter-in-Place, and (3) reported past 30-day use of any nicotine or cannabis (ie, tetrahydrocannabinol) e-cigarettes. The University of California, San Francisco Institutional Review Board approved this study. Supplemental eFigure 1 describes eligibility criteria and how the final analytic sample was derived (n = 85).

### Measures

Interest in quitting nicotine or cannabis e-cigarette use was a dichotomous outcome variable: those (very) interested in quitting versus those who were not or somewhat interested or “neutral.” The survey asked about reasons respondents had not quit e-cigarette use (eg, not considering these products harmful) and whether respondents were interested in learning about an Instagram-based nicotine or cannabis e-cigarette use cessation program (dichotomized from a 3-point scale: maybe interested or interested versus not interested).

We measured the 4-item nicotine or cannabis e-cigarette use dependence scale using the 4-item Electronic Cigarette Dependence Scale (EDS)^11^ as a proxy; a possible range was from 0 to 16, with higher values indicating stronger dependency (Cronbach α = 0.87). Sociodemographic characteristics included age, self-identified gender, race-ethnicity, and mother’s highest level of educational attainment (Supplemental eTable 1).

### Statistical Analysis

All analyses were conducted in SAS software, version 9.4 (SAS Institute, Cary, North Carolina). In unadjusted analyses, we used Pearson χ^2^ or Fisher exact tests for categorical variables and the Student *t* test and the Wilcoxon/Mann-Whitney *U* test for normally and non-normally distributed continuous variables, respectively. Results were deemed significant on alpha = 0.05 and marginally significant on alpha = 0.1. In adjusted analysis, controlling for sociodemographics (age, gender, race-ethnicity, and mother’s education), we carried out 6 multivariable penalized logistic regressions (a superior method to handle small sample and sparse data)^12^ with profile-likelihood confidence intervals (CIs) for nonlinear models^13^ to assess associations of interest in quitting nicotine or cannabis e-cigarettes with self-reported reasons for quitting that were significant in bivariate analyses.

As sensitivity analysis, we carried out 6 *traditional* multivariable logistic regression models with normal-based Wald CIs to compare results with the primary analysis that used penalized regression modeling with profile-based CIs (Supplemental eTable 2).

## Results

**Figure 1.**
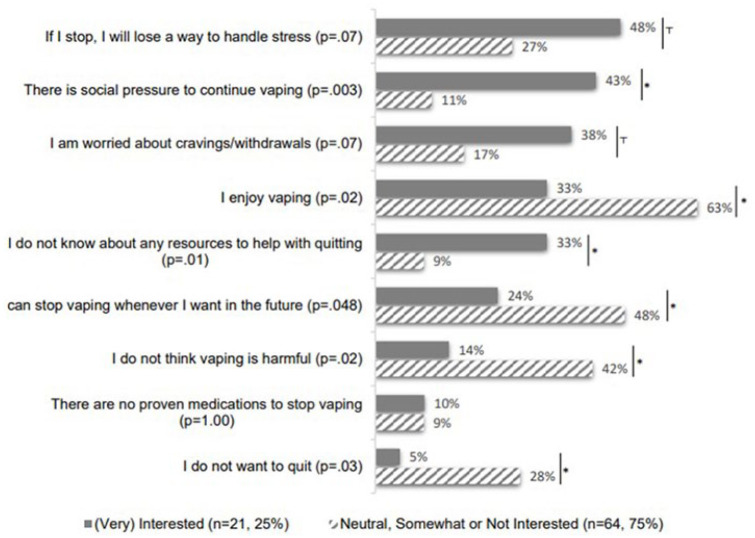
Self-reported reasons of not quitting vaping during COVID-19 Shelter-in-Place orders by interest in quitting nicotine/cannabis electronic cigarette use (n = 85). *P*-values were obtained from Pearson X^2^ or Fisher Exact tests. Percentages shown are rounded to the nearest whole number. *– statistically significant: *p* < 0.05; T– marginally significant: *p* < .1.

Among 85 respondents (mean age = 16.7 y.o.), 47% identified as female (Supplemental eTable 1), and 37 (44%) identified as Hispanic, aligning with other statewide California surveys.^14,15^ All respondents reported current (past 30-day) use of *nicotine* e-cigarettes; 68/85 (80.0%) reported current use of *cannabis* e-cigarettes. Almost a quarter (21/85, 24.7%) were interested in quitting nicotine or cannabis e-cigarettes, another 21/85 (24.7%) were neutral, 26/85 (30.6%) were somewhat interested and 17/85 (20.0%) were not interested. Most respondents (73/85, 85.9%) were eager to learn about an Instagram-based e-cigarette use cessation intervention, and 100% of those 21 who were interested in quitting expressed interest (Supplemental eTable 1). The top 3 reasons participants have not quit nicotine/cannabis e-cigarettes were “I enjoy vaping” (47/85, 55.3%), “I can stop vaping whenever I want in the future” (36/85, 42.4%), and “I do not think vaping is harmful” (30/85, 35.3%; Figure 1).

**Figure 2.**
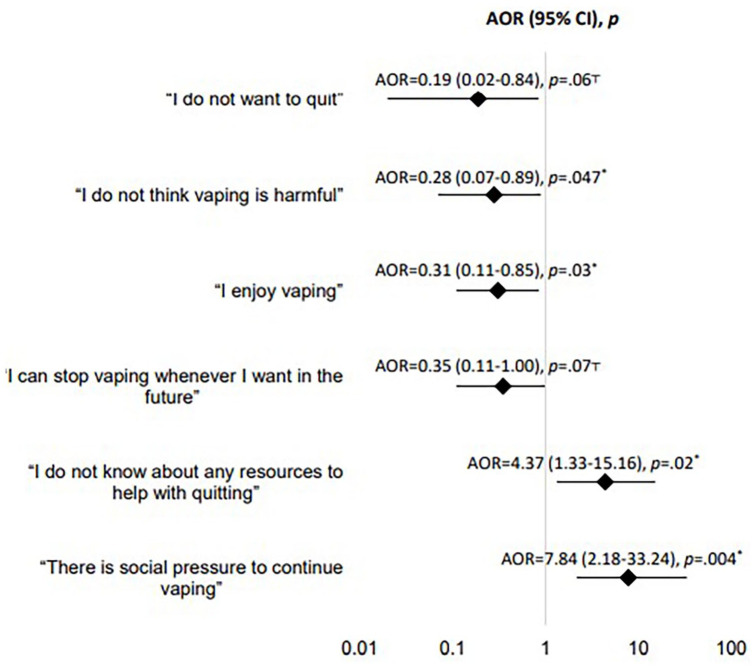
Self-reported reasons for not quitting nicotine/cannabis electronic cigarette use and adjusted associations with interest in quitting nicotine/cannabis e-cigarettes during COVID-19 Shelter-in-Place orders (n = 83)^a^. Interest in quitting nicotine/cannabis e-cigarette use was the outcome in all six penalized logistic regressions adjusted for age (in years), self-identified gender (male vs female), race-ethnicity (Hispanic; Other, non-Hispanic vs White, non-Hispanic participants), and mother’s highest educational attainment (Other vs College degree or higher). ^a^Complete case analysis was performed due to missing data (n = 1) and exclusion of those who responded “Non-binary/Another term” to the gender question (n = 1). *– statistically significant: *p* < 0.05; T – borderline statistically significant: *p* < .1. Abbreviations: AOR: Adjusted odds ratio.

Adjusting for sociodemographics, those interested in quitting nicotine or cannabis e-cigarettes were more likely to report feeling social pressure to continue use (adjusted odds ratio [AOR] = 7.84; 95% CI = 2.18-33.24, *P* = .004) and being unaware of resources to help with quitting (AOR = 4.37; 95% CI = 1.33-15.16, *P* = .02; Figure 2). Results from the sensitivity analysis to compare traditional versus penalized logistic regression models showed similar findings (Supplemental eTable 2).

## Discussion

Our exploratory cross-sectional study of 85 California adolescents who currently used nicotine or cannabis e-cigarettes revealed that almost a quarter were interested in quitting use during Shelter-in-Place, and 100% of those were intrigued by a cessation intervention on Instagram. Respondents interested in quitting nicotine or cannabis e-cigarettes were more likely to report continued use due to social pressure and low perceived risks of nicotine or cannabis e-cigarette products. The main reasons adolescents had not quit nicotine or cannabis e-cigarettes during Shelter-in-Place were enjoying e-cigarette use, no desire to quit at the moment but having confidence in ability to stop e-cigarette use in the future, and not considering nicotine or cannabis e-cigarettes harmful.

Consistent with our findings, prior research on e-cigarette cessation among adolescents and young adults (14-21 y.o.) also reported that many young people were interested in quitting e-cigarette products or reported at least one quit attempt,^16-18^ and considered digital platforms as a helpful prevention intervention tool.^18^ Although limited, emerging research demonstrates potential effectiveness of e-cigarette cessation interventions in youth (13-17 y.o.) via smartphone text messaging and successful recruitment through social media advertisement.^19^ The fact that 100% of adolescents in our study who were interested in quitting e-cigarette use were intrigued by a cessation intervention on Instagram speaks to the need for more authentic resources for help. Instagram is a social-networking site and mobile app popular among young people, with almost 160 million users in the United States.^20^ The first experimental study of Instagram-based intervention for electronic cigarette use cessation in youth has been completed and found the social media support group intervention improved abstinence from e-cigarette use in young people.^21,22^

Existing research has demonstrated negative health impacts of inhalation of aerosols regardless of the type of e-cigarette products (nicotine or cannabis),^23,24^ and the current evidence that e-cigarettes assist with smoking cessation is nonconclusive.^25,26^ Furthermore, a cohort study found that adolescent co-use of nicotine and cannabis e-cigarette products might increase the risk for nicotine dependence and the frequency of combustible cigarette smoking in young adulthood.^27^ Some studies report prevalent smoking and vaping cannabis among youth who are interested in nicotine e-cigarette cessation,^28^ with more desire to quit or reduce the use of nicotine e-cigarettes compared with cannabis products.^28,29^

Previous research has found that many young people (14-21 y.o.) interested in quitting e-cigarettes report health risks as the major reason to stop use;^18^ however, more than 35% of adolescents in our study did not perceive e-cigarette use as harmful. Given these findings, it is urgent for pediatricians and primary healthcare providers to educate patients and their parents or guardians on harms of nicotine or cannabis e-cigarettes among adolescents and to inform them about available resources to help with e-cigarette use cessation. Peer-counseling should also be considered in the development of intervention efforts. As adolescents’ lives move increasingly online,^10^ it is essential that e-cigarette education go digital as well. Parents, pediatricians, and public health professionals should also be aware of the links between social media consumption and increased usage of e-cigarette products.^30-33^

### Limitations

First, this descriptive study was a cross-sectional survey with a small convenience sample of California adolescents only. Potential for generalizability of the study results outside California may be limited; however, the demographic characteristics of the sample, with 44% of respondents of Hispanic ethnicity, aligns with California surveys^14,15^ that report from 47% to 52% of participants who identify as Hispanic. Second, data were self-reported and collected through online questionnaires during the COVID-19 lockdown period when students could have been monitored by their parents, guardians, or other adult relatives. Third, there is a risk of recall bias in questions that refer to e-cigarette use during the pre-COVID time. Fourth, we could not distinguish between the use of nicotine or cannabis e-cigarettes as all survey questions referred to the use of either product.

### Conclusions

Many participants were interested in quitting nicotine or cannabis e-cigarettes during Shelter-in-Place and the majority were interested in an Instagram-based cessation intervention. Given many participants have not quit nicotine or cannabis e-cigarettes due to low perceived risks and social pressure to continue use, future interventions should consider peer-counseling, aim to engage pediatricians and primary healthcare providers to increase awareness among adolescents, their parents, or guardians of harmful health effects of nicotine or cannabis e-cigarettes, and promote resources to assist with quitting.

## Author Contributions

*Vira Pravosud*: contributed to conception; contributed to analysis and interpretation of data; drafted the manuscript. *Pamela M. Ling*: contributed to conception; contributed to interpretation of data; critically revised manuscript. *Bonnie Halpern-Felsher*: contributed to conception; contributed to interpretation of data; critically revised manuscript. *Valerie Gribben*: contributed to conception and design; contributed to acquisition and interpretation of data; critically revised manuscript. All authors gave final approval and agreed to be accountable for all aspects of the work in ensuring that questions relating to the accuracy or integrity of any part of the work are appropriately investigated and resolved.

## Supplemental Material

sj-docx-1-cpj-10.1177_00099228251355748 – Supplemental material for Adolescents’ Interest in Quitting Nicotine or Cannabis E-Cigarettes Supplemental material, sj-docx-1-cpj-10.1177_00099228251355748 for Adolescents’ Interest in Quitting Nicotine or Cannabis E-Cigarettes by Vira Pravosud, Pamela M. Ling, Bonnie Halpern-Felsher and Valerie Gribben in Clinical Pediatrics
