# Supplementary material for: Adolescents’ Interest in Quitting Nicotine or Cannabis E-Cigarettes
Source: Clin Pediatr (Phila). 2025 Jul 20;64(11):1505–10. doi: 10.1177/00099228251355748 (PMC12489162; doi:10.1177/00099228251355748)
Supplement: sj-docx-1-cpj-10.1177_00099228251355748 – Supplemental material for Adolescents’ Interest in Quitting Nicotine or Cannabis E-Cigarettes [file sj-docx-1-cpj-10.1177_00099228251355748.docx]

**Supplemental Materials**

Contents

[**eFigure 1.** Flow diagram of the final sample selection 2](#_Toc197638019)

[**eTable 1.** Characteristics of respondents by interest in quitting nicotine/cannabis electronic cigarette use during COVID-19 Shelter-in-Place orders (n=85) 3](#_Toc197638020)

[**eTable 2.** Self-reported reasons for *not* quitting nicotine/cannabis electronic cigarette use and adjusted associations with interest in quitting nicotine/cannabis e-cigarettes during COVID-19 Shelter-in-Place orders (n=83) 4](#_Toc197638021)

Entries received: N=126

Eligibility criteria:

(1) 13-18 years old (SIS^a^ verified by supporting documentation),

(2) a middle or high school student in California before Shelter-in-Place, and

(3) reporting past 30-day use of any nicotine or tetrahydrocannabinol (THC) electronic cigarette product.

Invalid entries excluded based on manual check: n=29:

(1) demographic data (age, gender, and race),

(2) duration of survey completion,

(3) geolocation, and

(4) quality of responses to open questions.

Valid entries: n=97

Duplicate observations with shorter duration of survey completion: n=12^b^

Final analytic sample: n=85

# **eFigure 1.** Flow diagram of the final sample selection

^a^SIS International Research recruited adolescents by reaching out to parents on SIS research panels and by posting the screener questionnaire online. The survey questionnaires were pilot-tested and were designed to take 20 minutes to compete. Participants received a $20 gift card upon completion of valid surveys.

^b^The respondents’ names were kept confidential, and each participant was assigned a unique survey identification number to prevent multiple entries.

# **eTable 1.** Characteristics of respondents by interest in quitting nicotine/cannabis electronic cigarette use during COVID-19 Shelter-in-Place orders (n=85)

| **Variable** | **Not Interested, Somewhat Interested, or Neutral (n=64, 75.3%)** | **(Very) Interested (n=21, 24.7%)** | ***p*** |
| --- | --- | --- | --- |
| **Socio-demographic characteristics** |  |  |  |
| Age in years (n=84) | 16.69 ±1.2;  16.6 (16.1-17.7) | 16.7 ±1.4;  16.7 (15.9-17.6) | .93 |
| Self-identified Gender |  |  | .85^a^ |
| Female | 30 (46.9) | 9 (42.9) |  |
| Male | 33 (51.6) | 12 (57.1) |  |
| Other/non-binary | 1 (1.6) | 0 (0.0) |  |
| Self-identified Race-Ethnicity |  |  | .28^a^ |
| African American or Black and non-Hispanic | 9 (14.1) | 4 (19.0) |  |
| White and non-Hispanic | 18 (28.1) | 8 (38.1) |  |
| Another race and non-Hispanic^b^ | 9 (14.1) | 0 (0.0) |  |
| Hispanic^c^ | 28 (43.8) | 9 (42.9) |  |
| Mother’s highest level of education |  |  | .69^a^ |
| GED/High school or below | 19 (29.7) | 6 (28.6) |  |
| (Some) College degree | 27 (42.2) | 10 (47.6) |  |
| (Some) Graduate/Prof degree | 15 (23.4) | 3 (14.3) |  |
| Unknown | 3 (4.7) | 2 (9.5) |  |
| **Nicotine and/or cannabis electronic cigarette use during SIP and interest in cessation** | | | |
| Frequent e-cigarette use *during* SIP | 30 (46.9) | 9 (42.9) | .75 |
| E-cigarette dependency scale (0->16)^d^ | 8.3 ±4.1;  7.0 (4.0-11.0) | 8.5 ±5.4;  10.0 (6.0-13.0) | .81 |
| (Maybe) Interested in learning about an Instagram-based e-cigarette cessation program | 52 (81.3) | 21 (100.0) | .03* |

Numbers shown are frequencies (percentages) for categorical variables and mean ±standard deviation; median (25^th^-75^th^ percentiles) for continuous variables. P-values were obtained from Pearson χ^2^ or Fisher Exact tests for categorical variables and from the Student t-test (for normally distributed age) and the Wilcoxon/Mann-Whitney U test for non-normally distributed (e-cigarette dependency scale) continuous variables.

^a^Fisher Exact test used

^b^Includes one respondent of White race and unknown ethnicity.

^c^In our sample, 37/85 (44%) identified as Hispanic, which aligns with prior California surveys in 2018^15^ and 2020^16^ that included 47% and 52% participants of Hispanic ethnicity, respectively.

^d^Used as a proxy for nicotine/cannabis e-cigarette use dependence scale.

*– statistically significant: p<.05; ^┬^– marginally significant: p<.1

Abbreviations: GED: General Education Development Test; SIP: Shelter-in-Place

# **eTable 2.** Self-reported reasons for *not* quitting nicotine/cannabis electronic cigarette use and adjusted associations with interest in quitting nicotine/cannabis e-cigarettes during COVID-19 Shelter-in-Place orders (n=83)

| **Variable** | **Penalized**^a^ | | **Traditional**^a^ | |
| --- | --- | --- | --- | --- |
|  | AOR (95% CI) | **p** | AOR (95% CI) | ***p*** |
| 1. I do not want to quit | 0.19 (0.02-0.84) | 06^┬^ | 0.12 (0.02-1.00) | .050^┬^ |
| 1. I do not think vaping is harmful | 0.28 (0.07-0.89) | .047* | 0.23 (0.06-0.90) | .03^*^ |
| 1. I enjoy vaping | 0.31 (0.11-0.85) | .03^*^ | 0.27 (0.09-0.81) | .02^*^ |
| 1. I can stop vaping whenever I want in the future | 0.35 (0.11-1.00) | .07^┬^ | 0.35 (0.11-1.00) | .049^*^ |
| 1. I do not know about any resources to help with quitting | 4.37 (1.33-15.16) | .02^*^ | 5.12 (1.41-18.62) | .01^*^ |
| 1. There is social pressure to continue vaping | 7.84 (2.18-33.24) | .004^*^ | 10.40 (2.40-45.05) | .002^*^ |

Interest in quitting nicotine/cannabis e-cigarette use was the outcome in all logistic regressions (six penalized and six traditional) adjusted for age (in years), self-identified gender (male vs female), race-ethnicity (Hispanic; Other, non-Hispanic vs White, non-Hispanic participants), and mother’s highest educational attainment (Other vs College degree or higher).

^a^Complete case analysis was performed due to missing data (n=1) and exclusion of those who responded “Non-binary/Another term” to the gender question (n=1).

*– statistically significant: p<.05; ^┬^– marginally significant: p<.1

Abbreviations: AOR: Adjusted odds ratio
